# Heat and Mass Transfer of the Droplet Vacuum Freezing Process Based on the Diffusion-controlled Evaporation and Phase Transition Mechanism

**DOI:** 10.1038/srep35324

**Published:** 2016-10-14

**Authors:** Zhijun Zhang, Jingxin Gao, Shiwei Zhang

**Affiliations:** 1 School of Mechanical Engineering and Automation, Northeastern University, Shenyang 110004, China

## Abstract

A frozen phase transition model is developed to investigate the heat and mass transfer of a single water droplet during the vacuum freezing process. The model is based on the diffusion-controlled evaporation mechanism and phase transition characteristics. The droplet vacuum freezing process can be divided into three stages according to the droplet states and the time order. It includes the evaporation freezing stage, the isothermal freezing stage and the sublimation freezing stage. A numerical calculation is performed, and the result is analysed. The effects of the vacuum chamber pressure, initial droplet diameter and initial droplet temperature on the heat and mass transfer characteristics at each stage are studied. The droplet experiences supercooling breakdown at the end of the evaporation freezing stage before the isothermal freezing stage begins. The temperature is transiently raised as a result of the supercooling breakdown phenomenon, whose effects on the freezing process and freezing parameters are considered.

Droplet vacuum freezing is a fast temperature-change process. It is accompanied by phase transitions from liquid to vapour, liquid to solid and solid to vapour^1^. The heat and mass transfer during the droplet vacuum freezing process has become the research hotspot of the spray crystallization field^2^,^3^,^4^. It is essential to study its mechanism and influence factors for ice particle production^5^.

In the droplet vacuum freezing process, a single water droplet is frozen rapidly under vacuum conditions. During the freezing process, heat is absorbed from the droplet to support the evaporation or sublimation. The droplet temperature will decrease until the droplet is frozen into an ice particle^6^,^7^,^8^. The freezing process can be divided into three stages - the evaporation freezing stage, the isothermal freezing stage and the sublimation freezing stage - according to the droplet states and the time order. The droplet states in the three stages are entirely liquid, liquid–solid and entirely solid, respectively^9^. At the first stage, the evaporating freezing stage, the pressure of the droplet surface is far higher than the vacuum pressure. The fast evaporation on the surface of the droplet begins, and then the droplet temperature is decreased, the droplet diameter is reduced, and droplet mass is lost. The evaporation freezing stage will only be terminated after the temperature of the droplet is deeply below the equilibrium freezing temperature when it reaches the nucleation temperature^10^. It starts to form crystal nuclei at this temperature. The supercooling breakdown phenomenon of the droplet will appear before the isothermal freezing stage begins^2^,^9^. The temperature of the droplet rises back to the equilibrium freezing temperature immediately because of the supercooling breakdown phenomenon. This is called the effect of transient temperature rising. It occurs for the simultaneous phase transition of the droplet from liquid phase to solid phase. The phase transition heat is released in this process. Then, the isothermal freezing stage begins. The phase transition heat will be used to sustain the freezing process. Specific heat is not absorbed from the droplet itself, and the temperature is maintained during this stage. The isothermal freezing stage ends when the phase transition heat is depleted. Subsequently, the droplet temperature will continue dropping because of the sublimation phenomenon at the droplet surface^9^. This stage is called the sublimation freezing stage until the temperature reaches the final end temperature, which corresponds to the vacuum pressure.

Droplet vacuum freezing is a complicated process accompanied by heat and mass transfer. It has been studied by many researchers through numerical simulations and experimental research. Strub *et al*.^9^ studied the crystallization of a water droplet through experiments and modelling. In their study, the behaviour of water droplets sprayed out of a snow gun was modelled to investigate the heat and mass transfer processes of water droplets during the phase transition from the supercooled liquid phase to the liquid–solid phase and finally to the solid phase. Zhao *et al*.^3^ studied how the initial droplet diameter, initial droplet temperature and vacuum chamber pressure affect the heat and mass transfer during the freezing process by using the dynamic mesh method. The effect of the transient temperature rise was not considered. Shin *et al*.^7^ studied the forming conditions of ice particles by performing numerical simulation through the diffusion-controlled evaporation model to assess the feasibility of applying the water spray evaporation method to ice particle production. The diffusion-controlled evaporation model was well validated by comparing the simulation results with the experimental results. However, they did not consider the transient temperature rise effect. The finite element method has also been used to model the vacuum freezing of a single puree droplet, which was based on the heat and mass diffusion equations^4^.

Droplet vacuum freezing has practical applications in the fields of spray freeze drying, ice storage and so on refs 8 and 11. In this paper, a frozen phase transition model is developed based on the diffusion-controlled evaporation mechanism and phase transition characteristics. The model is used to investigate the heat and mass transfer processes of a single water droplet during the vacuum freezing process. The effects of vacuum chamber pressure, initial droplet diameter and initial droplet temperature are studied. The improvement of the frozen phase transition model is that the influence of the transient temperature rise effect on the freezing process and freezing parameter change is considered.

## Results and Discussion

The effects of the vacuum chamber pressure, initial droplet temperature and initial droplet diameter on the freezing parameters during the droplet vacuum freezing process are studied by using the frozen phase transition model in this paper.

### Effect of the vacuum chamber pressure on the freezing process

**Figure 1:**
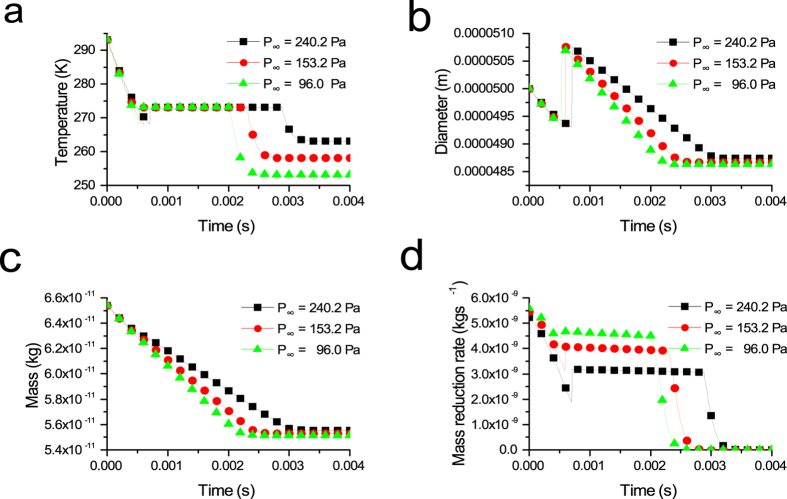
The variation of (**a**) droplet temperature, (**b**) droplet diameter, (**c**) droplet mass losses and (**d**) droplet mass reduction rate with different vacuum chamber pressures (D_P0_ = 50 μm, T_a0_ = 293.15 K).

The relationship between the pressure and temperature can be described as Eq. (5). Before the simulation begins, it is assumed that the final end temperatures of the three droplets are 263.15 K, 258.15 K and 253.15 K. Thus, the pressures of the droplet surfaces should be 240.2 Pa, 153.2 Pa and 96.0 Pa, respectively, when the droplet reaches the final end temperature according to Eq. (5). The lower the vacuum chamber pressure is, the lower the final end temperature of the droplet. The variations in the temperature, diameter, mass and mass reduction rate with different vacuum chamber pressures are shown in Fig. 1.

Figure 1a shows the effect of different vacuum chamber pressures on the droplet temperature. The lower the vacuum chamber pressure, the faster the temperature drops in the evaporation freezing stage, and the isothermal freezing stage is correspondingly quicker. Lowering the vacuum chamber pressure is effective in reducing the freezing time, which is very meaningful in the frozen stage.

The variation of the droplet diameter is shown in Fig. 1b, which indicates that the lower the vacuum chamber pressure is, the faster the droplet diameter decreases. The variation of the diameter with time is approximately linearly correlated in the isothermal freezing stage. The final percentages of diameter variations corresponding to 240.2 Pa, 153.2 Pa and 96.0 Pa are 2.52%, 2.65% and 2.75%, respectively. These results indicate that the droplets with lower vacuum chamber pressures have smaller diameters under the same conditions.

As shown in Fig. 1c, the changing relation of the droplet mass with time shows a downward trend. The percentages of the final mass losses of droplets frozen under pressures of 240.2 Pa, 153.2 Pa and 96.0 Pa are 15.1%, 15.5% and 15.7%, respectively. In addition, Fig. 1d shows that the mass reduction rate decreases faster under higher vacuum chamber pressure. It also indicates that the higher is the vacuum chamber pressure is, the less droplet mass is lost.

### Effect of the initial droplet temperature on the freezing process

**Figure 2:**
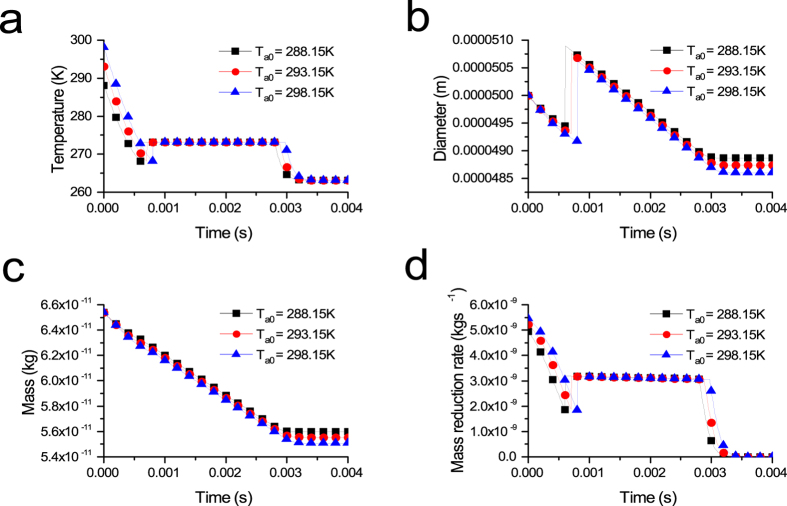
The variation of (**a**) droplet temperature, (**b**) droplet diameter, (**c**) droplet mass losses and (**d**) droplet mass reduction rate with different initial droplet temperatures (D_P0_ = 50 μm, P_∞_ = 240.2 Pa).

The variations of the temperature, diameter, mass and mass reduction rate with different initial droplet temperatures are shown in Fig. 2.

The variation of the droplet temperature with different initial droplet temperatures is shown in Fig. 2a. The three curves of the evaporation freezing stage are nearly parallel, which demonstrates that the temperature reduction rates of the droplets with different initial droplet temperatures are almost the same in this stage. As shown in Fig. 2a, the lower the initial droplet temperature is, the shorter the freezing process. The evaporation freezing stage and the sublimation freezing stage are affected by the initial droplet temperature. However, it has little influence on the freezing time of the isothermal freezing stage. That difference between the freezing times of the isothermal freezing stage is 0.00003 s when the difference in the initial droplet temperature is 10 K. In addition, the initial droplet temperature does not affect the final end temperature of the droplet; it affects only the time the droplet spends to reach the steady state.

Figure 2b shows the changing curves of droplet diameter under different initial droplet temperatures. In the evaporation freezing stage, the higher the initial droplet temperature is, the more rapidly the droplet diameter decreases. Droplets with higher initial temperatures spend more time in the evaporation freezing stage and incur larger mass losses than those with lower initial temperatures. Thus, the droplet whose initial temperature is 298.15 K had the smallest diameter after the jump phenomenon of the diameter. In addition, raising the initial droplet temperature can cause more mass loss of the droplet, as shown in Fig. 2c.

Figure 2d shows the variation of the mass reduction rate with different initial droplet temperatures. The changing trend of the mass reduction rate is similar among them. The droplets with high initial temperatures have faster mass reduction rates in the evaporation freezing stage and the sublimation freezing stage. Their mass reduction rate in the isothermal freezing stage is nearly the same. The entire mass reduction rate finally decreases to zero.

### Effect of the initial droplet diameter on the freezing process

**Figure 3:**
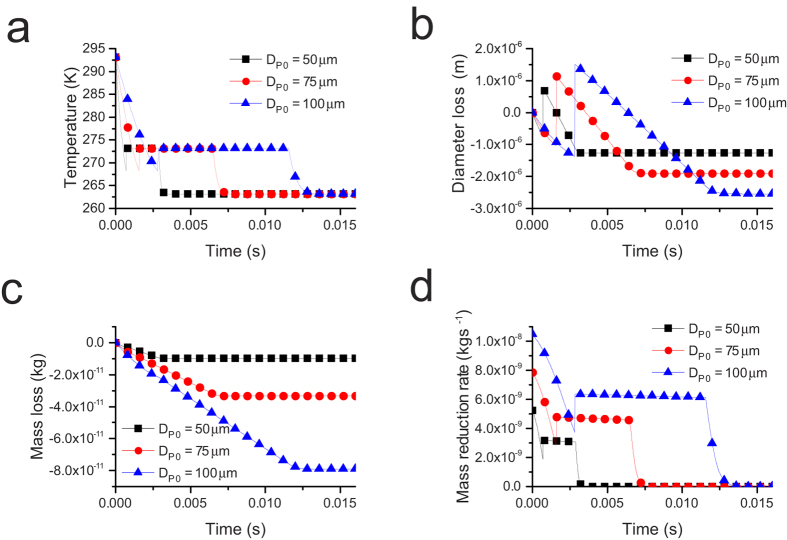
The variation of (**a**) droplet temperature, (**b**) droplet diameter loss, (**c**) droplet mass loss and (**d**) droplet mass reduction rate at different initial droplet diameters (P_∞_ = 240.2 Pa, T_a0_ = 293.15 K).

The variations of the temperature, diameter, mass and mass reduction rate with different initial droplet diameters are shown in Fig. 3. The end temperature of droplets with different initial diameters is the same under these conditions.

As shown in Fig. 3a, all droplet temperatures decrease rapidly in the evaporation freezing stage. However, the temperature of the droplets with smaller sizes drops more rapidly than those with larger sizes. The freezing time of the isothermal freezing stage is also shorter than the others. It is obvious that the smaller the initial droplet diameter is, the shorter the freezing process. That is, large droplets need more time to freeze completely. Because fast freezing is ideal in most situations, small droplets are better choices for this purpose.

As shown in Fig. 3b,c, the larger the droplet diameter is, the higher the variation of the droplet diameter and the droplet mass loss. However, the calculation results show variations of the droplet diameters of 2.52%, 2.53% and 2.54% corresponding to diameters of 50 μm, 75 μm and 100 μm, respectively, and the percentages of the mass losses of the three droplets are nearly the same, which suggests that the initial droplet diameter has a very small effect on the variation of droplet diameter and droplet mass loss. Therefore, controlling the droplet diameter will not be a useful method to reduce the mass loss.

Figure 3d shows that the initial droplet diameter obviously influences the mass reduction rate. Smaller droplets have lower mass reduction rates at the beginning of the freezing process. The mass reduction rate of the smaller droplets also dropped more rapidly than the others.

## Conclusions

The frozen phase transition model is developed to investigate the parameter variation and the heat and mass transfer of a single water droplet at each stage during the vacuum freezing process. The model is based on the diffusion-controlled evaporation mechanism and phase transition characteristics. The simulation results of the frozen phase transition model are compared with the simulation results and experimental results from the literature. The effects of the vacuum chamber pressure, the initial droplet diameter and the initial droplet temperature on the freezing process are studied in this paper. After detailed analysis and discussion, the improvements of the frozen phase transition model are presented in this paper. The frozen phase transition model can accurately illustrate the real droplet vacuum freezing process. The important influence of the transient temperature rise of the droplet on the freezing process is proved by the frozen phase transition model.

The lower the vacuum chamber pressure is, the smaller the diameter and the greater the mass loss of the droplet after the freezing process ends. The end temperature will also be lower. The end temperature depends only on the vacuum chamber pressure. In addition, lowering the vacuum chamber pressure is effective in reducing the freezing time. Raising the initial droplet temperature has less influence on the end temperature but can increase the mass loss under the same conditions. The effect of the initial droplet diameter on the freezing parameters is quite small. Larger droplets need more time to freeze completely. The mass loss of a droplet during the freezing process is mainly determined by the vacuum chamber pressure and the initial temperature. Thus, in the droplet vacuum freezing process, the reduction of the droplet mass loss can be achieved by increasing the vacuum chamber pressure or lowering the initial temperature.

## Model and Method

### Frozen phase transition model

**Figure 4:**
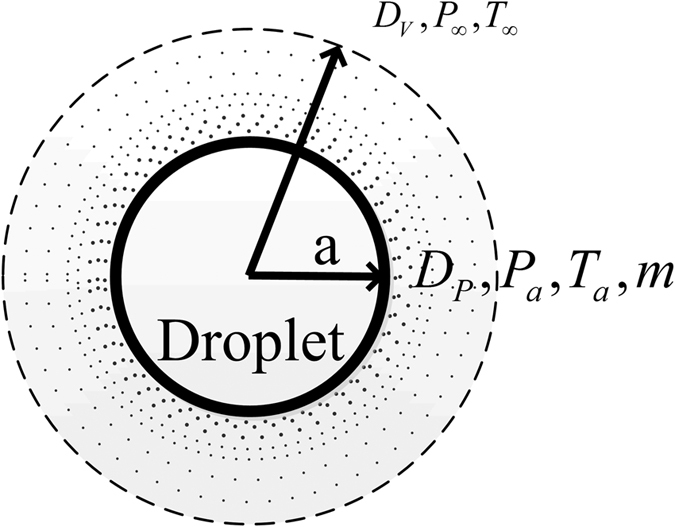
Evaporation phenomenon on the droplet surface.

The diffusion-controlled evaporation model is used to explain the evaporation phenomenon on the droplet surface during the droplet vacuum freezing process, as shown in Fig. 4. Shin *et al*.^7^ explained this model clearly. Based on the research results by Shin *et al*., this paper presents an improved model named frozen phase transition model. The three stages of the droplet vacuum freezing process and the influence of the transient temperature rise effect caused by the supercooling breakdown phenomenon are comprehensively studied.

The droplet diameter is very small, and the freezing process of the droplet is rapid. The freezing process of the droplet is affected by many factors, including the droplet size and freezing conditions^3^,^9^. It is difficult to predict the nucleation temperature and the end temperature of the crystallization process because of these features. Strub *et al*.^9^ designed simulations to study the influence of the initial droplet diameter, air relative humidity, air temperature and crystallization temperature on the freezing process. The results from their study showed that most nucleation temperatures of droplets were between 269.15 K and 265.15 K, and the final end temperature of the freezing process was within the range of 263.15 K to 259.15 K under the experimental conditions they designed. Hindmarsh *et al*.^2^ also found that the nucleation temperature of a droplet changed with the simulated experimental conditions. According to the features of the frozen phase transition model (FPTM), the nucleation temperature is set to 268.15 K in the present study. The correlation equations of the frozen phase transition model are described as follows, and more details about the diffusion-controlled evaporation mechanism can be found throughout the literature^7^,^8^.

Assuming that the water vapour can be regarded as an ideal gas, the mass reduction rate of the droplet can be described as follows:


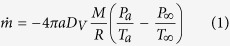


where 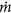 is mass reduction rate of droplet or mass flowrate of vapour (kg/s), *a* is radius of droplet (m), *D*_*V*_ is diffusion coefficient of vapour (m^2^/s), *R* is universal gas constant, (J/(mol K)), *P*_*a*_ is the pressure of droplet surface (Pa), *P*_∞_ is the pressure in chamber (Pa), *T*_*a*_ is the temperature of droplet (K), *T*_*∞*_ is the temperature of chamber (K).

The mass of the droplet is


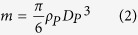


where *ρ*_*P*_ is the droplet density (kg/m^3^), *D*_*P*_ diameter of droplet (m).

Integrating Eq. (1) and Eq. (2) gives


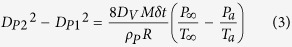


where *D*_*P*1_ is initial diameter of droplet (m), *D*_*P*2_ is diameter of droplet after *δt* (m), *δt* is timestep (s).

The droplet surface temperature is used to represent the centre droplet temperature in the frozen phase transition mode. The variation of the droplet temperature can be obtained by


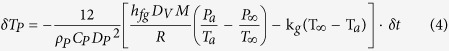


where *δT*_*p*_ is temperature variation of droplet (K), *C*_*P*_ is specific heat (J/(kg K)), *h*_*fg*_ is the latent heat of vaporization (J/kg), *k*_*g*_ is thermal conductivity, (W/(m K)).

The vapour pressure variation of the droplet surface is expressed by


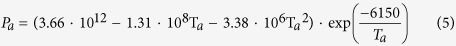


The temperature transiently rises because of the supercooling breakdown phenomenon. It is an exothermic process. The heat released by this process is *Q*_*p*_, and the heat used for increasing the temperature is *Q*_*c*_. The droplet then enters the isothermal freezing stage; this stage lasts Δt and needs Δ*Q* to maintain the temperature constant. The corresponding formulas of this stage are expressed as follows.

The heat used by the droplet temperature increase process can be described as


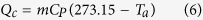


The heat release for the phase transition is determined by


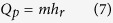


where *h*_*r*_ is latent heat of phase transition (J/kg).

The time of the isothermal freezing stage is calculated by the following equation:


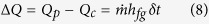


### The calculation flowchart

**Figure 5:**
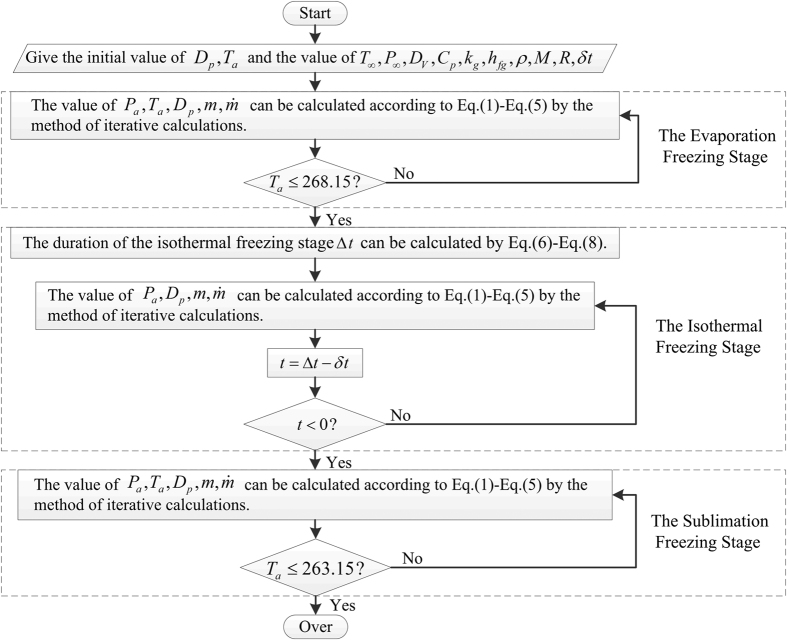
The calculation flowchart of the droplet vacuum freezing process.

The calculation flowchart of the droplet vacuum freezing process is shown as follows (Fig. 5). According to Eq. (5), the value of the vacuum chamber pressure corresponding to the boiling point temperature of 263.15 K is 240.2 Pa.

### Validation

**Figure 6:**
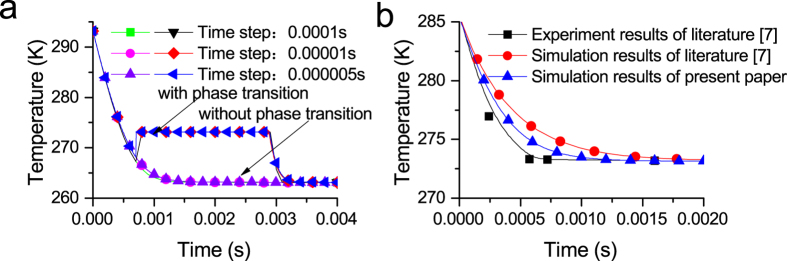
Temperature variation of a droplet with (**a**) different time steps, and (**b**) simulation results of the frozen phase transition model, and the simulation and experimental results from the literature^7^ (D_P0_ = 50 μm, P_∞_ = 240.2 Pa, T_a0_ = 293.15 K).

The time step independence was verified using different time steps. The droplet diameter is 50 μm. Its initial temperature is 293.15 K. The pressure of the vacuum chamber is 240.2 Pa (corresponding to 263.15 K). The results are shown in Fig. 6.

Figure 6a shows that it is reasonable to set the time step to 0.00001 s in the calculation of the droplet vacuum freezing process. Therefore, the time step was unified to 0.00001 s in the following calculations.

**Figure 7:**
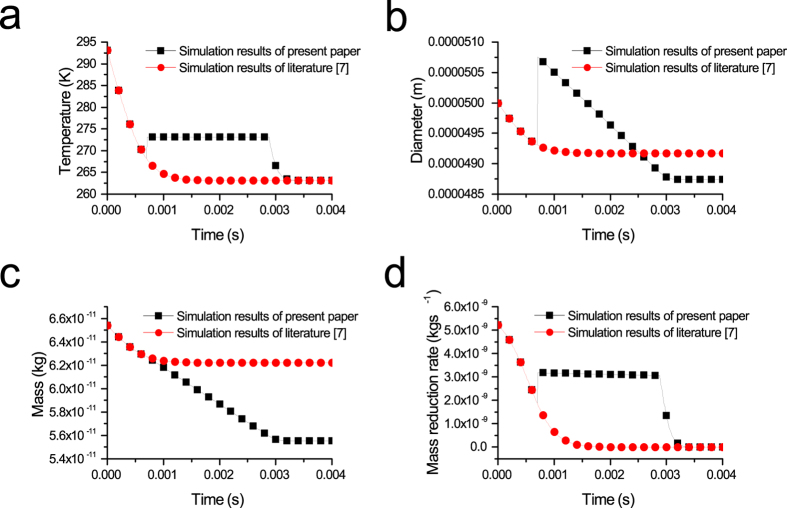
(**a**) Temperature variation, (**b**) diameter variation, (**c**) mass variation and mass reduction rate variation of the frozen phase transition model versus the literature^7^ (D_P0_ = 50 μm, P_∞_ = 240.2 Pa, T_a0_ = 293.15 K).

Figure 6b shows the results of the frozen phase transition model, without considering the transient temperature rise effect, and the simulation and experimental results from the literature^7^. The frozen phase transition model has been improved based on the model from the literature^7^, but phase transition does not occur in Fig. 7 because the temperature is higher than the phase transition temperature. The simulation result of the frozen phase transition model is closer to the experimental curve than the simulation results from the literature^7^. The experimental curve is obtained by fitting the five experimental points from the literature^7^. The difference in the results is due to the different choices in the associated physical property parameters.

### The improvements of the frozen phase transition model

The frozen phase transition model considers the influence of the transient temperature rise effect on the freezing parameters, which is the major improvement of the frozen phase transition model. The difference in the frozen phase transition model and reference methods are shown in Fig. 7.

The simulation results from the literature^7^ show that the curves of temperature, diameter, mass and mass reduction rate declined smoothly during the entire freezing process and are different from the results of the frozen phase transition model.

The comparison result of the droplet temperature variation is shown in Fig. 7a. In the evaporation freezing stage, both of the droplet temperatures drop rapidly, and there is no obvious difference between the two simulation results. After the evaporation freezing stage ends, the temperature variation result from the literature^7^ continued to decline until the freezing process ends. The droplet temperature of the frozen phase transition model rises immediately before the isothermal freezing stage begins because of the supercooling breakdown phenomenon. The droplet temperature then remains unchanged in the isothermal freezing stage until starting to decline again in the sublimation freezing stage. The freezing process ends when the droplet temperature reaches the final end temperature.

The variations of the droplet diameter and the droplet mass are shown in Fig. 7b,c, respectively. The jump phenomenon of the droplet diameter occurs at the moment the isothermal freezing stage begins. The droplet diameter then continues to decrease until the freezing process ends. The sudden increase in droplet diameter is due to the phase transition from liquid phase to solid phase during the transient temperature rising process. The droplet density decreases, but the mass remains unchanged. The final droplet diameter of the frozen phase transition model is smaller than the final droplet diameter from the literature^7^. The droplet mass variation curves both have decreasing patterns. The difference between them is the percentage of droplet mass loss. The percentage of droplet mass loss of the frozen phase transition model is much greater than the result from the literature^7^, which is due to the phase transition heat causing greater loss of mass.

Figure 7d shows the mass reduction rate of the droplet during the freezing process. The changing trend of the mass reduction rate of the frozen phase transition model is similar to the temperature variation of the frozen phase transition model in Fig. 7a, which is due to the mass reduction rate calculated by Eq. (1). The decrease in the mass reduction rate means that the mass loss of the droplet during the freezing process becomes increasingly slower. Both mass reduction rates finally decreased to zero.

Moreover, the freezing time of the frozen phase transition model is longer than the result from the literature^7^ according to Fig. 7. This result is due to the existence of the phase transition heat.

The influence of the transient temperature rise effect was not considered on the freezing process for the simulation results from the literature^7^. The changes caused by the supercooling breakdown phenomenon are not considered. The droplet vacuum freezing process is significantly affected by the transient temperature rise and phase transition. Thus, the frozen phase transition model, obtained by a significant improvement to the model from the literature^7^, can much better reflect the real freezing process.

## Additional Information

**How to cite this article**: Zhang, Z. *et al*. Heat and Mass Transfer of the Droplet Vacuum Freezing Process Based on the Diffusion-controlled Evaporation and Phase Transition Mechanism. *Sci. Rep.* **6**, 35324; doi: 10.1038/srep35324 (2016).

## References

[b1] Zhang Z., Zhang Y., Zhao L., Zhang W. & Zhao S. Parameter Sensitivity of the Microdroplet Vacuum Freezing Process. Math. Probl. Eng. Article ID 370159 (2015).

[b2] Hindmarsh J. P., Russell A. B. & Chen X. D. Experimental and numerical analysis of the temperature transition of a suspended freezing water droplet. Int. J. Heat. Mass. Transf. 46, 1199–1213 (2003).

[b3] Zhao L., Zhang Y., Zhang Z., Li X. & Zhang W. Heat and Mass Transfer of Droplet Vacuum Freezing Process Based on Dynamic Mesh. Math. Probl. Eng. Article ID798040 (2014).

[b4] Cogné C., Nguyen P. U., Lanoisellé J. L. & Hecke E. V. D. Clausse, Modeling heat and mass transfer during vacuum freezing of puree droplet. Int. J. Refrig. 36, 1319–1326 (2013).

[b5] Bédécarrats J. P., David T. & Lasvignottes J. C. Ice slurry production using supercooling phenomenon. Int. J. Refrig. 33, 196–204 (2010).

[b6] Feuillebois F., Lasek A., Creismeas P. & Szaniawski A. Freezing of a Subcooled Liquid Droplet. J. Colloid Interf. Sci. 169, 90–102 (1995).

[b7] Shin H. T., Lee Y. P. & Jurng J. Spherical-shaped ice particle production by spraying water in a vacuum chamber. Appl. Therm. Eng. 20, 439–454 (2000).

[b8] Kim B. S., Shin H. T., Lee Y. P. & Jurng J. Study on ice slurry production by water spray. Int. J. Refrig. 24, 176–184 (2001).

[b9] Strub M., Jabbour O., Strub F. & Bédécarrats J. P. Experimental study and modelling of the crystallization of a water droplet. Int. J. Refrig. 26, 59–68 (2003).

[b10] Satoh I., Fushinobu K. & Hashimoto Y. Freezing of a water droplet due to evaporation-heat transfer dominating the evaporation–freezing phenomena and the effect of boiling on freezing characteristics. Int. J. Refrig. 25, 226–234 (2002).

[b11] Kozawa Y., Aizawa N. & Tanino M. Study on ice storing characteristics in dynamic-type ice storage system by using supercooled water: Effects of the supplying conditions of ice-slurry at deployment to district heating and cooling system. Int. J. Refrig. 28, 73–82 (2005).

